# God and the Possibility of a Moral Right to Privacy

**DOI:** 10.1007/s11841-024-01057-3

**Published:** 2025-02-08

**Authors:** Björn Lundgren

**Affiliations:** 1 Centre for Philosophy and AI Research, Friedrich-Alexander-Universität Erlangen-Nürnberg, Erlangen, Germany; 2 Institute for Futures Studies, Stockholm, Sweden; 3 Department of Philosophy, Stockholm University, Stockholm, Sweden

**Keywords:** Privacy, The right to privacy, The right to privacy and God

## Abstract

In their *Unfit for the Future*, Ingmar Persson and Julian Savulescu argued that there is no moral right to privacy, which resulted in a string of papers. This paper addresses their most recent contribution, arguing that—contrary to their claims—there is no conflict between God and a moral right to privacy.

In their *Unfit for the Future*, Ingmar Persson and Julian Savulescu (2012) argue that there is no moral right to privacy, which resulted in a string of papers (Bublitz, 2019; Lundgren, 2021; Persson & Savulescu, 2019, 2022). Here I respond to their most recent clarifications, which in turn respond to a paper by Björn Lundgren. Granted that Persson and Savulescu ignore most of Lundgren’s arguments, I will not aim to defend them; instead, I will address one of their latest arguments, which will yield some insight into the potential conflict between God and a right to privacy.

In this paper, I will first contribute to this debate by elucidating an important part of the underlying metaethical and metaphysical presumptions in their argument. Indeed, I will show that their argument is only sound, if it is sound at all, in case God actually or possibly exists. Second, given this result and because many people believe that God exists, I will turn to argue that the existence of God would not imply a problem for the possibility of a moral right to privacy because God does not violate anyone’s right to privacy.

Given that Persson and Savulescu’s argument is less clear than what is normally desirable I will quote directly from them:

Many people believe that human beings have a moral right to privacy. A significant portion of those who have this belief are probably Christians who believe in God. But God, as conceived by Christians, is commonly thought to be an omniscient, omnipotent and omnibeneficent or morally perfect being. If we have rights to privacy, the conception of such a being would be inconsistent, for by being omniscient, it would violate the right to privacy of countless human (and non-human?) beings and, thus, would not be morally perfect. The divine being has acquired as much information about us as is conceivable; yet it has not violated our alleged right to privacy. How is that possible if we have such a right? (2022: 2)

As I stated above, I will aim to settle some of the underlying metaphysical and metaethical presumption before we engage with the normative argument. Although we should perhaps presume that Persson and Savelescu take a secular perspective,^1^ I will argue that the only plausible paths to make this argument worthwhile depends on arguing that God exists either actually or possibly.

Thus, let me first set aside two alternative interpretations of their argument that does not rely on any presumption of God’s existence. The first option is that their argument is purely conceptual. Simply put, the argument might be that the standard conception of God shows that there cannot be a moral right to privacy. However, if that is their argument, then we can establish that there are no moral rights *simpliciter* because any moral right would conflict with some conceivable—or describable—agent. Such an argument is arguably absurd, and therefore an argument against a moral right to privacy cannot be based purely on the conceptual nature of God.

The second option that they grant that God is impossible, but if God did exist, then a moral right to privacy would be impossible. Although there are technical proposals on how impossibilities can be used to inform us about metaphysical facts, it is not clear exactly how this argument would function. Indeed, if we reason from impossibilities, then it seems that we can draw the contrary conclusion as well.

Hence, given that we have set aside a conceptual reading and an interpretation that denies God’s possible existence, it means that we are left with the implication that God must exist (either actually or possible), if Persson and Savulescu’s argument is to have any standing.

Starting with God’s actual existence, under this interpretation, the argument requires that one can show that God exists (i.e., if there is supposed to be a conflict between God’s actual existence and the right to privacy, the former must be established). Since no God-proof is provided by Persson and Savulescu and since no God-proof has proven broadly acceptable so far, the argument fails to establish a necessary premise.

An alternative is to lessen the modal claim, and suppose that God’s existence is merely possible. However, this modal claim must also be defended. Moreover, even if it may be simpler to prove God’s possible existence, there are further problems. Persson and Savulescu have not explained what role possible agents have in determining moral truths. That is, one must specify the modal status of the possibility claim and one must make clear how that affects our moral rights. Although many think that normative truths hold in all possible worlds, others (e.g., Fine, 2002), think that normative necessities are distinct from metaphysical and natural necessities. More importantly, few think that the right to privacy is morally robust (see, e.g., Véliz, 2024 for an exception, but see also Munch, forthcoming for a counterexample), but that takes us into the more applied normative arguments, so we can set that aside for now (as you will see, we need not address specific conceptions of the right to privacy to resolve Persson and Savulescu’s argument).^2^

I am not expecting Persson and Savulescu to resolve all queries about modality and existence and how modal claims about the existence of agents should influence our rights and duties, but we get no indication at all of their view on this. Contrary to the controversiality of these issues they claim that “[t]he thought-experiment of an omniscient being strikes us as being as close to conclusive as anything you might hope to find that there is no right to privacy” (2022: 4). In conclusion, their argument depends on some sort of God proof (either in terms of God’s possible or actual existence). Hence, let us suppose God’s existence and ask whether it implies that our right to privacy is violated. There are several separate reasons why we should *deny* the idea that God violates our moral right to privacy.

First, we might think that the right to privacy is a right only against other humans, not a right against God. Although limiting ethical theories to purely related to humans’ actions can be accused of a problematic anthropocentrism, it is not unreasonable to think that certain rights are about human interactions. Indeed, just as someone might think that a dog cannot diminish or affect their privacy one may think that God cannot either.^3^ One reason to hold such a view would be that privacy is an interpersonal property and God (or nonhuman animals) are distinctly different in a way such that human privacy cannot be affected by their information acquisition. Persson and Savulescu provide no argument to explain why human privacy and the right thereof should be affected by any nonhuman entities. Referring to the fact that God would know everything about us tells us nothing about whether the right to privacy can be violated by nonhuman lifeforms or entities. Any argument that aims to deny a moral right to privacy because God is omniscient must explain why we should think that God can violate our right to privacy.

Second, even if we think that God’s omniscience diminishes our privacy, it does not follow that our right to privacy has been violated. Standardly, a privacy right violation requires wrongdoing, which means that someone’s privacy can be diminished without any wrongdoing (e.g., if share something privacy-sensitive with someone or they accidentally hear it). Under normal circumstances, this other person has not violated my right to privacy.

In the case of God, there are several reasons why we might think that there is no wrong-doing involved (cf., e.g., Kahane, 2011). One reason may be that God, in virtue of necessarily being omniscient, cannot avoid knowing everything about us. If God cannot avoid knowing everything, then we standardly would think that there is no wrong-doing involved. However, in response to such an argument, Persson and Savulescu argue that agents such as God “should be able to curtail its omniscience should its goodness demand it” (2022: 4). However, if God has that ability (and I would happily grant this for the sake of argument), then we could argue that God would respect our right to privacy unless it is overridden by other concerns (cf. Falls-Corbitt & McLain, 1992). Indeed, if God can curtail his omniscience, then we should presume that God, being all-good, would act in a way such that he curtails his omniscience whenever not doing so would violate someone’s right to privacy. Thus, all such an argument can show is that the right to privacy is overridable. But that is hardly surprising, nor is it controversial.

While more can be said, this suffices to show that Persson and Savulescu simply fail to establish their conclusion. First, any argument that aims to establish that there is no moral right to privacy because of God, depends on proof of God’s actual or possible existence (and the latter requires further grounding). Conceptual arguments or arguments from impossibility, will not suffice. Moreover, even if God exists, it is not clear that God’s existence establishes any problem for a moral right to privacy. Indeed, as I have argued, all they can hope to show is that our right to privacy is overridable.

However, there is another form of challenge that may be worth considering. In a parallel to the problem of evil, we may argue that just as it is a problem that God—if God exists—lets evil happen, it would be a problem if God lets moral rights be violated.^4^ While situations when God diminishes our privacy (if that is can be the case), can be explained as a non-violation because a moral right to privacy is overridable (and God would only infringe upon the right when it is overridable), the same argument might not apply to every human action. However, that seems to be a problem for *any* moral right. For example, consider a human killing another, innocent, human. We cannot claim that all such cases are cases when the slain human’s right to life is overridden. Yet, God is clearly in a position to alter any such infringements. Hence, we have a potential conflict. However, it is difficult to see how that conflict provides any reason to deny the existence of any moral rights, generally, or a moral right to privacy specifically. Just as *the problem of evil* is viewed as a challenge against God’s existence (which requires explaining)—not as an argument against the existence of evil—it would be similarly odd to claim that the potential conflict between God and moral rights implies a denial of the possibility of moral rights. Hence, I find no reason to worry about a moral right to privacy because of any form of existence of God.^5^

## Data Availability

N/A.

## Footnotes

1 As suggested by one of the reviewers.

2 One might *prima facie* think that this is an odd argument on my part, as we often make use of possibilities in philosophical arguments. Indeed, we rarely presume that thought experiments are—or need be—*actually* true. In this case, we have set aside the interpretation according to which the Persson and Savulescu’s argument presumes God’s actual existence. This leaves the reading of God’s possible existence open for further interpretations. For example, one might hold that God could not actually come to exist, but rather that God’s existence is possible in the sense that the actual world could have been otherwise in a way such that God would have actually existed. This differs from many (but not all) thought experiments that are temporally possible (i.e., even if they are not actualized at this moment or in the past, they could happen in the future). The relevance of the status of the possibility claim relates to a much larger debate about the modal robustness of our normative theories that I will not engage with further in this paper; all I want to highlight here is that there are some complications that Persson and Savulescu have ignored.

3 Although Pepper (2020) recently argued that humans violate a right to privacy held by animals, this does not imply that human’s privacy can be violated by animals or by God.

4 Although it is not clear whether the problem of evil has addressed the potential conflicts with moral rights (see, e.g. Tooley 2021 for an overview), there is a parallel between God letting evil happen and letting moral rights be violated.

5 A further complicating factor may be so-called *positive rights*. Presuming a standard Hohfeldian analysis of rights as implying duties, and for simplicity that rights are grounded in individuals’ interests, positive rights obligate actions that contributes to the individuals’ specific interest, while negative rights merely make certain actions, which interfere with that interest, impermissible. As such, there could be a further problem of God not performing the duties following from positive rights. This might be viewed as somewhat different from the problem of evil as it cannot be explained, for example, by a difference between action and inaction. However, the argument is not about a moral right to privacy as such, but an argument against *positive moral rights* as such. Moreover, just as the ordinary problem of evil (in which God is, apparently, letting evil things happen), we should say that this is a problem for explaining how God’s existence is compatible with God neglecting his duties (i.e., if the duties implied from positive rights are duties for God).

## References

[CR1] Bublitz, J. C. (2019). Saving the world through sacrificing liberties? A critique of some normative arguments in unfit for the future. *Neuroethics,**12*, 23–34.

[CR2] Falls-Corbitt, M., & McLain, M. (1992). God and privacy. *Faith and Philosophy.,**9*(3), 369–386.

[CR3] Fine, K. (2002). The Varieties of Necessity. In T. Gendler & J. O’Leary-Hawthorne (Eds.), *Conceivability and possibility* (pp. 253–281). Clarendon Press.

[CR4] Kahane, G. (2011). Should we want God to exist? *Philosophy and Phenomenological Research,**82*, 674–696.

[CR5] Lundgren, B. (2021). Why extending actions through time can violate a moral right to privacy. *Journal of Ethics and Social Philosophy,**20*, 111–118.

[CR6] Munch, L. A. (Forthcoming). Why the NSA didn’t diminish your privacy but might have violated your right to privacy. *Analysis*. Pre-print available at: https://philpapers.org/archive/MUNWTN.pdf

[CR7] Pepper, A. (2020). Glass panels and peepholes: Nonhuman animals and the right to privacy. *Pacific Philosophical Quarterly,**101*, 628–650.

[CR8] Persson, I., & Savulescu, J. (2012). *Unfit for the future: The need for moral enhancement*. Oxford University Press.

[CR9] Persson, I., & Savulescu, J. (2019). The irrelevance of a moral right to privacy for biomedical moral enhancement. *Neuroethics,**12*, 35–37.

[CR10] Persson, I., & Savulescu, J. (2022). The impossibility of a moral right to privacy. *Neuroethics,**15*, 23. 35784396 10.1007/s12152-022-09500-3 PMC9239928

[CR11] Tooley, M. (2021). The problem of evil. *The Stanford Encyclopedia of Philosophy* (Winter 2021 ed).

[CR12] Véliz, C. (2024). *The ethics of privacy and surveillance*. Oxford University Press.

